# When to Achieve Complete Revascularization in Infarct-Related Cardiogenic Shock

**DOI:** 10.3390/jcm11113116

**Published:** 2022-05-31

**Authors:** Giulia Masiero, Francesco Cardaioli, Giulio Rodinò, Giuseppe Tarantini

**Affiliations:** Department of Cardiac, Thoracic, Vascular Sciences and Public Health, University of Padua Medical School, Via Giustiniani 2, 35128 Padova, Italy; giulia.masiero.86@gmail.com (G.M.); francesco.cardaioli@gmail.com (F.C.); giulio.rodino92@gmail.com (G.R.)

**Keywords:** cardiogenic shock, acute myocardial infarction, mechanical circulatory support, left ventricular assist devices

## Abstract

Acute myocardial infarction (AMI) complicated by cardiogenic shock (CS) is a life-threatening condition frequently encountered in patients with multivessel coronary artery disease (CAD). Despite prompt revascularization, in particular, percutaneous coronary intervention (PCI), and therapeutic and technological advances, the mortality rate for patients with CS related to AMI remains unacceptably high. Differently form a hemodynamically stable setting, a culprit lesion-only (CLO) revascularization strategy is currently suggested for AMI–CS patients, based on the results of recent randomized evidence burdened by several limitations and conflicting results from non-randomized studies. Furthermore, mechanical circulatory support (MCS) devices have emerged as a key therapeutic option in CS, especially in the case of their early implantation without delaying revascularization and before irreversible organ damage has occurred. We provide an in-depth review of the current evidence on optimal revascularization strategies of multivessel CAD in infarct-related CS, assessing the role of different types of MCS devices and highlighting the importance of shock teams and medical care system networks to effectively impact on clinical outcomes.

## 1. Introduction

Cardiogenic shock (CS) is a life-threatening condition predominantly caused by coronary artery disease (CAD), which occurs in 5–10% of patients presenting with acute myocardial infarction (AMI), with a higher incidence in ST-segment elevation MI (STEMI) compared with non-STEMI (NSTEMI) [1,2,3]. Early coronary revascularization, particularly percutaneous coronary intervention (PCI), has had the greatest impact on CS prognosis [4]. However, despite recent therapeutic and technological advances, the mortality rate for patients with CS related to AMI remains high, with an in-hospital rate value of 40–60% [5,6]. The presence of multivessel CAD (MVD) is a risk factor for CS development and can be found in up to 50% of acute coronary syndromes (ACS) [7,8]. Adjunctively, incomplete revascularization (both anatomical and functional) has been identified as a strong independent predictor of cardiovascular outcomes in ACS [9,10], outside the CS setting. Randomized evidence and meta-analyses have widely proven a decreased risk of composite outcomes by achieving a complete revascularization in hemodynamically stable AMI patients affected by MVD regardless of the mode of selection and the timing of the non-culprit lesion treatment [9,10]. Conversely, the recent CULPRIT-SHOCK trial demonstrated a clinical benefit from a culprit lesion-only (CLO) revascularization compared to an immediate multivessel PCI in patients with CS, which is actually contraindicated according to the latest guidelines [11]. Nevertheless, several limitations of this trial have been described, together with conflicting results from non-randomized studies [9,10,12]. Furthermore, mechanical circulatory support (MCS) devices have emerged as a key therapeutic option for CS patients, especially in the case of early implantation without delaying revascularization and before irreversible organ damage has occurred [13]. The objective of this manuscript was to provide an in-depth review of the current evidence on optimal revascularization strategies of multivessel CAD in infarct-related CS, assessing the effective impact of MCS, shock teams and medical care system networks on clinical outcomes.

## 2. Definitions of Complete Revascularization

The presence or absence of untreated residual CAD after treatment (with coronary artery by-pass graft, CABG, or PCI) defines the completeness of revascularization and has important prognostic implications [14]. In the past years, several different definitions of complete revascularization have been proposed, based either on the extent of residual anatomic disease (i.e., the presence of residual critical lesions) or on the degree of residual jeopardized ischemic myocardium due to untreated lesions, as evidenced by both non-invasive and invasive functional tests [14,15,16]. At present, two main definitions of complete revascularization are commonly used:

- Anatomic complete revascularization, usually defined as successful treatment of all lesions with a diameter stenosis ≥50% or ≥70% in vessels with a reference diameter ≥1.5/2.0 mm, with slight differences in cut-off values among different studies. Other authors refer to anatomic complete revascularization when a residual SYNTAX score of 0 is achieved. This latter definition provides a more objective and standardized parameter which was linked to a better post-procedural outcome prediction [17].
- Ischemic (i.e., functional) complete revascularization, defined as successful treatment of all flow-limiting lesions, responsible for either resting or stress-induced ischemia or pathological fractional flow reserve values [14,15].

When the criteria for complete revascularization are not met, incomplete revascularization is present, defined as “reasonable” when functional but not anatomic complete revascularization is achieved [9].

## 3. Cardiogenic Shock Complicating Acute Coronary Syndromes

CS is a progressive adverse condition characterized by severe impairment of myocardial performance leading to reduced cardiac output and systemic hypoperfusion [18,19]. Several criteria for the diagnosis of CS exist, all of which include hypotension (systolic blood pressure <90 mmHg despite adequate filling status or administration of vasopressors to maintain systolic blood pressure >90 mmHg) accompanied by symptoms and signs of end-organ hypoperfusion (such as cold sweated extremities, oliguria, mental confusion, dizziness and narrow pulse pressure) in the setting of myocardial impairment [20]. Of note, hypoperfusion is not always accompanied by hypotension, as blood pressure may be preserved by compensatory vasoconstriction (with/without pressor agents) [21].

A large spectrum of clinical statuses, ranging from pre-shock to refractory shock states, can characterize CS patients [19,20,21,22]. Recently, the Society for Cardiovascular Angiography and Interventions (SCAI) consensus statement on the classification of CS proposed a useful classification for defining this spectrum of disease severity, with five evolving stages of shock defined as A to E (Table 1) [23].

A variety of conditions, both acute and chronic, can be related to CS, such as AMI, severe valvular diseases or end-stage chronic heart failure. Moreover, though left ventricular systolic failure is the most common cause of reduced pump function, CS can be also driven by right ventricular or bi-ventricular disfunction [24].

ACS are the most frequent cause of CS, representing more than 70% of the cases [1]. Moreover, patients affected by STEMI have the greatest risk of developing CS (with approximately a 10% incidence [25]), compared to those affected by both unstable angina and NSTEMI. Despite recent improvements in therapeutic options (such as the use of MCS devices and advanced monitoring systems), the morbidity and mortality associated with CS remain high, with multiple pathophysiologic pathways involved in the deterioration of patients’ status [26]. In fact, while STEMI–CS is associated with massive and localized impaired coronary flow with rapid myocardial necrosis, NSTEMI–CS is usually characterized by diffuse flow-impairment with gradual myocardial injury [27]. These different characteristics also lead to different treatment strategies which are usually more aggressive and timelier in STEMI patients [28].

Moreover, among patients who developed CS, those without ST-segment elevation had more frequently several adverse baseline characteristics than those with ST-segment elevation, such as significantly older age, and a greater frequency of prior infarction, multivessel disease and congestive heart failure [29,30,31].

## 4. Early Revascularization in Infarct-Related Cardiogenic Shock

The management of CS should start as early as possible because of the reversible effects of tissue hypoperfusion (cellular and tissue hypoxia resulting in cellular death) in early stages, while a delay in diagnosis and treatment usually leads to irreversible changes, resulting in multi-organ failure and death [31]. Previous data have shown a short-lived window of opportunity to attempt to avert the development of CS, with a median time of 11 h from the beginning of symptoms and an irreversible shock stage [32]. Once the diagnosis of AMI–CS is confirmed by the identification of clinical signs of hypoperfusion, biochemical imbalance, and imaging/instrumental demonstration of myocardial ischemia/infarction [20], a timely reperfusion of the infarct-related artery must be performed, in the setting of both STEMI and NSTEMI. The randomized SHOCK (Should We Emergently Revascularize Occluded Coronaries for Cardiogenic Shock) trial demonstrated that, in patients presenting with AMI–CS, an early revascularization strategy (by means of PCI or CABG) improved the long-term survival compared to initial intensive medical therapy: all-cause mortality at 6 months was lower in the group assigned to revascularization than in the medically treated patients (50.3 vs. 63.1%, respectively; RR 0.80, 95% CI 0.65–0.98, *p* = 0.03) [1]. Furthermore, the early revascularization strategy resulted in a 13.2% absolute and a 67% relative improvement in 6-year survival [33]. Interestingly, a subsequent sub-analysis demonstrated a significant interplay between patient age and timing of revascularization. Indeed, the worse outcomes observed in older patients compared to younger patients (<75 years old), were mainly due to a higher mortality among those treated later, while subjects who underwent an early revascularization had comparable outcomes irrespective of age [34]. Similar results were reported in several observational studies describing the positive relationship existing between an early revascularization treatment and survival in both NSTEMI and STEMI patients [35,36]. Accordingly, current international clinical guidelines strongly recommend emerging coronary angiography and PCI of the culprit lesion for patients with cardiogenic shock due to STEMI or NSTE–ACS, independent of the time delay of symptom onset, if coronary anatomy is amenable to PCI (Class I, LOE B); otherwise, emergency CABG is the recommended alternative, especially for patients with severe and diffuse CAD with no obvious culprit lesion and/or with late-onset symptoms and considering that surgical treatment is a more traumatic and time-demanding procedure [37]. These recommendations have led to the wide implementation of the early revascularization strategy in patients with ACS complicated by CS, with a subsequent reduction in short-term mortality rate (40–50% nowadays) [11,38,39].

Besides a prompt revascularization, initial medical management with invasive hemodynamic assessment and respiratory support remains of pivotal importance, as it allows performing the required invasive procedures under acceptable hemodynamic conditions [20]. Inotropic and vasopressor agents are administered in up to 90% of AMI–CS patients as the first line of treatment for hypotension and hypoperfusion, but they may increase left ventricle afterload and may as well worsen myocardial ischemia and arrhythmias [20,31].

## 5. Revascularization Strategies of Multivessel CAD in AMI–CS Patients

Observational data showed that more than 70% of patients presenting with ACS and CS have significant stenoses in at least one non-infarct-related artery [4,6,40,41,42,43]. Moreover, it has been demonstrated that MVD is an independent predictor of in-hospital mortality [42,43], as the presence of multiple significant lesions in the context of CS may lead to a diffuse myocardial ischemia involving not only the culprit artery but also non-infarct-related lesions. This may occur through a pan-myocardial inflammatory process combined with systemic hypotension, leading to further coronary hypoperfusion in the non-infarct-related arteries and creating a vicious circle of further myocardial ischemia and impaired myocardial function [44]. However, the optimal management of multiple significant stenoses in a CS setting remains challenging. On one hand, a complete coronary revascularization should improve cardiac perfusion and output; on the other hand, a multivessel PCI may be associated with increased procedural time and higher procedural complications and contrast-induced nephropathy risks [12].

Outside of the CS setting, complete revascularization of non-culprit lesions (both anatomical and ischemic), when achievable, is the treatment of choice for patients with MVD presenting with STEMI [37] or NSTE–ACS [45]. The current recommendation for a routine delayed (staged) revascularization in patients presenting with STEMI (Class IIa, LOE A) has recently been strengthened by the large, multicenter, randomized COMPLETE trial, which showed a significative reduction of a composite clinical endpoint in patients treated with an anatomic complete revascularization strategy compared with a CLO strategy at 3-year follow-up, mainly driven by a reduction in myocardial infarction and ischemia-driven revascularization [46]. Key randomized trials comparing revascularization strategies in patients with AMI and MVD are listed in Table 2.

Differently from the hemodynamically stable setting, in the case of AMI–CS, the current guidelines contraindicate the routine immediate revascularization of non-culprit lesions during PCI in patients presenting with both STEMI and NSTEMI (Class III recommendation) [37,45]. These recommendations are based on the results of the CULPRIT SHOCK trial: in this large, randomized, multicenter trial involving 706 patients with MVD and AMI–CS, CLO-PCI (with the option of staged revascularization of non-culprit lesions) was superior to immediate multivessel PCI (MV-PCI) with respect to a composite endpoint of death or need for renal replacement therapy at 30 days [45.9% vs. 55.4%, RR 0.83 (0.71–0.96), *p* = 0.01]. The difference was mainly driven by significantly lower all-cause death in the CLO-PCI group [11]. At 1-year follow up, in the immediate MV-PCI group, mortality still tended to be higher, with no difference between rates at 30 days and 1 year, but with a lower occurrence of heart failure rehospitalization and repeat revascularization [52]. Importantly, the SYNTAX score was an independent predictor of adverse outcomes, with higher absolute risk with left main or proximal left anterior descending involvement and with no interaction between the SYNTAX score and the revascularization strategy [53]. Several limitations of these study have been described. First of all, half population underwent resuscitation before PCI, and the excess mortality in the MV-PCI arm was driven by anoxic brain damage [9,54]. Second, a non-negligible rate of cross-over between groups occurred (12.5% in the CLO-PCI group and 9.4% in the other arm) [55]. Third, CTO patients were largely (23%) represented in the study, and CTO PCI was performed in the acute MV-PCI group with a non-negligible rate of procedural unsuccess (19%). This may have contributed to increasing the duration and degree of complexity of the revascularization procedures in this group of patients, despite the lack of proven benefit of CTOs recanalization for clinical outcomes even in hemodynamically stable patients [12]. Lastly, the use of MCS systems was left to operator discretion, and this resulted in low employment rates (28%, of which 12% were microaxial pumps) [56,57].

Nevertheless, a recent CULPRIT SHOCK substudy analyzed the extent and severity of CAD following PCI by assessing the residual SYNTAX score (rSS), a scoring system that had already demonstrated its prognostic relevance in AMI patients [17,58,59]. The authors revealed that, despite the adoption of a MV-PCI strategy, complete revascularization was achieved in only one-fourth of the patients, mostly comprising younger patients with low CAD burden. Furthermore, the residual Syntax Score was strongly correlated with the basal score, reflecting the extent and severity of CAD in AMI–CS and highlighting the technical difficulty of achieving optimal revascularization in patients with multivessel and complex CAD. After multiple adjustments, the residual Syntax Score was confirmed as an independent predictor of 30-day mortality (adjusted odds ratio per 10 units: 1.49; 95% CI: 1.11 to 2.01) and 1-year mortality (adjusted odds ratio per 10 units: 1.52; 95% CI: 1.11 to 2.07) also in a CS setting [60]. Despite not having the strength to overturn them, these findings partly clash with the results of the original CULPRIT-SHOCK trial, highlighting the role of management of the non-culprit lesion as a key determinant of prognosis. Indeed, in the CLO-PCI arm of the CULPRIT SHOCK trial, planned non-culprit lesion stenosis PCI was performed in about one-half of the surviving patients [11,60]. Although underpowered to draw conclusive evidence, the results of this analysis may suggest that after a safe and effective CLO-PCI strategy in the acute phase of CS (avoiding inappropriate high-risk and unprotected PCI attempts), a staged revascularization to reduce rSS may benefit the patients [59].

Data from several national registries were retrospectively analyzed according to the CULPRIT SHOCK trial criteria: the findings of these studies were consistent also with respect to the evidence of the CULPRIT SHOCK trial [61,62,63]. Conversely, two large retrospective studies analyzed only AMI–CS patients with MVD presenting with STEMI and showed that, after propensity-score matching and multivariable regression, MV-PCI was associated with a significative lower risk of all-cause death and non-IRA repeat revascularization at long-term follow-up [64,65,66]. Notably, in these studies, CR was defined according to the anatomic criterion, and non-culprit lesion revascularization was not performed in the CLO-PCI groups. Furthermore, similarly to the CULPRIT SHOCK trial, low rates of MCS systems usage were reported. Therefore, rather than diverging from the results of the CULPRIT SHOCK trial, these studies once more highlighted a plausible impact of non-culprit lesions on long-term prognosis and the current need of further randomized controlled trials to better define patient selection and modalities and timing of complete revascularization in the setting of AMI–CS.

## 6. Role of MCS in AMI–CS Patients

In the last years, temporary MCS devices have emerged as a key therapeutic option for CS patients, especially for those with hemodynamic instability poorly controlled with standard medical therapy. MCS devices have, at present, a class IIa recommendation in the latest international guidelines for the treatment of acute HF [20]. As already demonstrated [67], an early and prompt MCS implantation without delaying revascularization and before irreversible organ damage has occurred, to restore proper coronary and end-organ perfusion is important to reach the greatest benefit possible from the device use. The most common hemodynamic support devices employed in AMI–CS patients are the intra-aortic balloon pump (IAPB, a cylindrical balloon placed in thoracic aorta, which actively deflates in systole and inflates in diastole), Impella (a co-axial pump, placed into the heart through a peripheral artery, that pumps blood from the LV to the ascending aorta), and extracorporeal membrane oxygenation (ECMO, an extracorporeal technique providing prolonged bi-ventricular and respiratory support). Despite optimal patient and device selection strategies are far from being defined, the assessment of acuity of illness, CS phenotype and vascular access anatomy remains essential to properly choose the type of support required (Table 3).

At present, only small observational studies have evaluated ECMO use in severe refractory AMI–CS patients, showing high mortality rate and a lower rate of in-hospital mortality, respiratory failure, and vascular complications compared to patients treated with Impella [68,69]. Despite the lack of randomized data, ECMO can be used as a bridge-to-recovery, bridge-to-bridge, and bridge-to-transplant for patients with refractory shock [20].

To date, data from RCTs have demonstrated a poor impact of IABP use on hard clinical endpoints [70]. The IABP Shock II Trial showed no benefits of IABP use compared to medical therapy alone on mortality at 30 days, 12 months and 6-year follow-up in patients with AMI–CS receiving early revascularization therapy [71,72,73]. Despite several limitations (no standardized timing for IABP insertion, absence of a standardized revascularization protocol, significative crossover rate between treatment groups), the trial results led to a class III recommendation for its routinary use in Europe [68] and a class IIb recommendation in the U.S.A. [74], suggesting its usefulness only in patients with mechanical complications after acute MI (Class IIb) [20].

The Impella pump received Food and Drug Administration approval for its use in CS patients in 2016, based on the results of the Recover I trial and the US Pella registry [75,76]. Despite a proven superior hemodynamic profile compared to IABP (driven by effective changes in myocardial oxygen demand, ventricular filling pressure and coronary perfusion) [13], to this day, the published randomized data failed in demonstrating differences in short- and long-term mortality between IABP and Impella in CS patients [77]. Furthermore, recent a retrospective analysis (hampered by a selection bias and heterogeneous treatment strategies) highlighted a greater rate of bleeding complications with the Impella system [72,77,78]. Based on these results, the current guidelines suggest the use of Impella as left ventricle unloading for ECMO-treated patients [20] or as bridge-to-decision therapy for carefully selected patients [79,80].

Despite the limiting recommendations, the use of Impella in AMI–CS patients, especially in those undergoing percutaneous revascularization, has been increasing over the last years. Data derived from several European and US registry studies, showed that the Impella system is the most commonly used device in AMI–CS patients, with an increase in use of nearly fivefold between 2012 and 2017 [57,81,82,83]. Subsequent analyses derived from these registries, identified the timing of implantation and the extension of revascularization as specific predictors of improved outcomes in patients undergoing mechanically supported PCI in a CS setting (Table 4).

Indeed, several non-randomized registry studies demonstrated better outcomes when the Impella support was initiated prior to PCI and applied in patients who had not experienced a cardiac arrest [67,76,84,85]. In a sub-study of the multicenter IMP-IT registry in AMI–CS patients [56], pre-PCI Impella insertion was independently associated with an improvement in survival (*p* < 0.01) and the composite of mortality, re-hospitalization for heart failure, and need for left ventricular assist device/heart transplantation at 1 year (*p* = 0.01) compared to later device insertion. Interestingly, an early implantation strategy was also associated with a significant lower rate of life-threatening bleeding (*p* = 0.02) and post-procedural acute kidney injury (AKI) (*p* = 0.04). Similarly, a retrospective multi-national analysis including Impella-treated AMI–CS patients showed a significant lower 30-day mortality rate when a pre-PCI device implantation was attempted (*p*= 0.003) [86]. The ongoing or future ANCHOR (Assessment of Extracorporeal Membrane Oxygenation in Acute Myocardial Infarction Cardiogenic Shock; NCT04184635), ECLS-SHOCK (Extracorporeal Life Support in Cardiogenic Shock; NCT03637205), EUROSHOCK (Testing the Value of Novel Strategy and Its Cost Efficacy in Order to Improve the Poor Out- comes in Cardiogenic Shock; NCT03813134), and DanGer Shock (Danish Cardiogenic Shock Trial; NCT01633502) trials will compare VA-ECMO or Impella use with standard guideline-directed therapy and will evaluate the validity of the suggested survival benefit with device placement before PCI.

Despite a class III recommendation for the routine revascularization of non-infarct-related lesions in AMI–CS patients [37], it is still a matter of debate whether the use of MCS devices may impact on the outcomes and improve the survival of MVD patients undergoing extensive revascularization during index procedure. A sub-analysis of the IMP-IT registry on CS showed a significantly lower occurrence of death and non-fatal MI at 1-year follow-up in patients undergoing more extensive revascularization (*p* = 0.001) [87]. The analysis from the NCSI (National Cardiogenic Shock Initiative; NCT03677180) trial demonstrated, in the setting of early MCS use, no difference in hospital survival between patients undergoing multivessel PCI versus those subjected to culprit-vessel PCI, as well as a similar incidence of post-procedural AKI [88], suggesting a possible beneficial role of early MCS implantation in MVD patients undergoing extensive revascularization. Finally, a recent observational study of AMI–CS patients treated with Impella-supported PCI in four high-volume European shock centers, showed a lower 30-day mortality among patients achieving complete revascularization (residual Syntax Score of 8 or less) compared to those characterized by incomplete revascularization (*p* = 0.009). Likewise, the most promising outcome was observed in patients with pre-PCI Impella implantation and complete revascularization compared to patients with post-PCI Impella implantation and incomplete revascularization (*p* < 0.001) [57]. Though routine extensive revascularization during index PCI procedure has been associated with worse 30-day outcomes, more data are needed to evaluate the possible beneficial impact of an early MCS implantation in these patients, allowing a safer and easier complete revascularization strategy.

## 7. Role of Shock Teams and Medical Care System Networks

To note, large contemporary registries observed unacceptable high rates of conservative management and delays to PCI in the infarct-related CS population [41,89]. In-hospital multidisciplinary shock teams in high-volume centers and regional systems of care are now proposed as a possible solution to improve prognosis. Indeed, although optimal protocols and structures for CS management have not been outlined so far, emerging data have suggested that the establishment of a shock team of multiple specialists (i.e., critical care cardiology, advanced HF cardiology, interventional cardiology, and cardiac surgery) might impact on CS outcomes [20,90,91]. Moreover, initial studies suggested that the implementation of a CS regional network with standardized referral protocols might improve patients’ survival, allowing a faster management of this critical life-threatening status [12]. All these efforts might allow a comprehensive multiorgan system care, improving early shock recognition and proper treatment.

## 8. Conclusions

**Figure 1:**
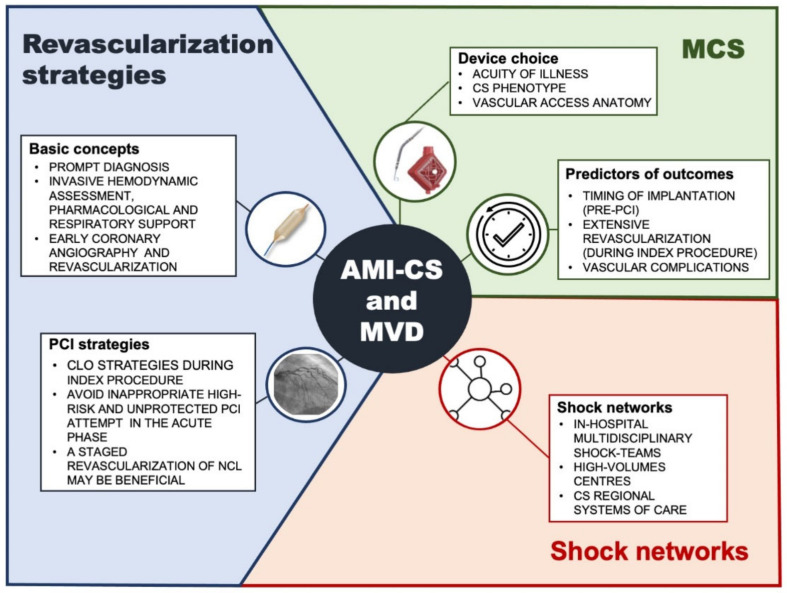
Current best practice for infarct-related cardiogenic shock patients with multivessel coronary artery disease: optimal revascularization strategies, mechanical cardiac support implantation, and shock teams and medical care system networks implementation. CS: cardiogenic shock; MCS: mechanical cardiac support; CLO: culprit-lesion only; NCL: non-culprit lesion; PCI: percutaneous coronary intervention.

Despite therapeutic and technological advances, CS complicating AMI in patients with MVD remains associated with unacceptably mortality and morbidity rates. The current best practice is based on a prompt diagnosis followed by a timely CLO revascularization with an unavoidable pharmacological and mechanical support (Figure 1). Emerging observational experience suggested that an early implantation of MCS prior to PCI, the performance of an extensive revascularization and the implementation of shock teams and medical care system networks are key factors for improving clinical outcomes (Figure 1). However, future randomized trials are warranted to determine the definitive role of a tailored complete revascularization (whether anatomical or functional) in an AMI–CS setting, both during a protected index PCI and in a staged procedure after the acute phase.

## Figures and Tables

**Table 1:** SCAI (Society for Cardiovascular Angiography and Interventions) stages of cardiogenic shock.

**Stage A** **(At Risk)**	At risk for cardiogenic shock (no signs or symptoms).
**Stage B** **(Beginning)**	Clinical evidence of relative hypotension or tachycardia without hypoperfusion (pre-shock).
**Stage C** **(Classic)**	Hypoperfusion with normal blood pressure or hypotension requiring intervention beyond volume resuscitation (inotropes, vasopressors, or mechanical support).
**Stage D** **(Deteriorating)**	Extreme hypoperfusion with hypotension or inotropes/vasopressors, failing to respond to initial interventions (similar to stage C and worsening).
**Stage E** **(Extremis)**	End-stage hypoperfusion with hypotension despite multiple interventions (inotropes/vasopressors/mechanical support).

**Table 2:** Overview of the key randomized control trials comparing revascularization strategies in patients with acute myocardial infarction and multivessel disease.

Trial Name/First Author	Clinical Characteristcs	Sample Size	Arms	Definition of NCL	Endpoints (Mace/Macce Rates)
PRAMI [47] 2013	STEMI	465	CVO PCI vs. MV primary PCI	%DS ≥ 50%	22.9% vs. 9.0% (*p* < 0.001) ^a^ at 23 months
CvLPRIT [48] 2015	STEMI	296	CVO PCI vs. MV primary or staged PCI	%DS > 70% in 1 view or >50% in 2 views	21.2% vs. 10.0% (*p* = 0.009) ^b^ at 12 months
DANAMI-3-PRIMULTI [49] 2015	STEMI	627	CVO PCI vs. MV staged PCI	%DS >50% with FFR ≤0.80	22.0% vs. 13.0% (*p* = 0.004) ^c^ at 27 months
SMILE [50] 2016	NSTEMI	542	Immediate MV PCI vs. MV staged PCI	Not reported	13.6% vs. 23.2% (*p* = 0.004) ^d^ at 1 year
COMPARE-ACUTE [51] 2017	STEMI	885	CVO PCI vs. MV primary or staged PCI	%DS ≥ 50% with FFR ≤0.80	20.5% vs. 7.8% (*p* < 0.001) ^e^ at 1 year.
CULPRIT SHOCK [11] 2017	Acute MI with cardiogenic shock	686	CVO PCI vs. MV primary PCI	%DS > 70%	45.9% vs. 55.4% (*p* = 0.01) ^f^ at 30 days
COMPLETE [46]	STEMI	4041	CVO PCI vs MV PCI either during or after the index hospitalization	%DS > 70% or DS > 50% with FFR ≤ 0.80	10.5% vs. 7.8% (*p* = 0.004) ^g^ at 3 years

NCL: non-culprit lesion; %DS, angiographic percentage of diameter stenosis; CVO, culprit vessel-only; FFR, fractional flow reserve; MACCE, major adverse cardiovascular and cerebrovascular events; MACE, major adverse cardiac events; MI, myocardial infarction; MV, multivessel; NSTEMI, non-ST segment elevation myocardial infarction; PCI, percutaneous coronary intervention; STEMI, ST segment elevation myocardial infarction. ^a^ MACE is a composite of cardiovascular death, non-fatal MI, refractory angina. ^b^ MACE is a composite of death, MI, any repeat revascularization and heart failure. ^c^ MACE is a composite of death, MI and any repeat revascularization of non-culprit vessel. ^d^ MACCE is a composite of cardiac death, MI, rehospitalization for unstable angina, TVR and stroke. ^e^ MACCE is a composite of death, MI, any repeat revascularization and cerebrovascular events. ^f^ MACE is a composite of death and renal-replacement therapy. ^g^ MACE is a composite of cardiovascular death and MI.

**Table 3:** Key factors that should be evaluated when choosing the type of mechanical cardiac support to restore proper coronary and end-organ perfusion in case of in infarct-related CS.

Patient’s Characteristics	Detailed Evaluation	MCS Selection
Acuity of illness	According to SCAI classification	Impella: may be used in stages C and D (in case of potentially reversible underlying cause or HT/VAD candidates). ECMO: may be used in stage C–E (especially in case of combined respiratory insufficiency or refractory cardiac arrest). IABP: routine use not recommended (may be limited in case of mechanical complications post-AMI)
CS phenotype	Type of cardiac failure	Impella: Isolated LV failure, biventricular injury without pulmonary failure (in combination with right p-VAD), isolated RV failure (no solid evidence). ECMO: biventricular injury (especially in case of combined respiratory insufficiency or refractory cardiac arrest), isolated RV failure (no solid evidence). IABP: routine use not recommended (may be limited in case of mechanical complications post-AMI)
Vascular access anatomy	Ilio-femoral/axillary access suitability	Impella CP and RP are implanted percutaneously, the larger devices are implanted surgically (according to cannula/sheath diameter) The femoral approach should be preferred A strict adherence to best vascular access and closure practices, familiarity with device troubleshooting and a multidisciplinary approach should be guaranteed.

CS: cardiogenic shock; SCAI: Society for Cardiovascular Angiography and Interventions; HT: heart transplantation; (p) VAD: (percutaneous) ventricular assist device; LV: left ventricle; RV: right ventricle; ECMO: ExtraCorporeal Membrane Oxygenation; IABP: intra-aortic balloon pump; AMI: acute myocardial infarction.

**Table 4:** Overview of the available studies investigating the impact of the timing of Impella 2.5/CP insertion and the extent of revascularization on outcomes in patients with AMI complicated by CS and treated with PCI.

**Timing of Impella Insertion**					
**Study**	**N° of pts.**	**N° of centers**	**Impella Devices**	**Study Groups**	**Main Findings**
Tarantini et al. [56]	147	17 Italian centers	2.5/CP	Insertion before PCI (*n* = 55) vs. during/after PCI (*n* = 92)	Pre-PCI insertion was associated with higher 1-year freedom from all-cause death [HR 0.45, CI (0.21–0.99); *p* =0.009], lower rates of in-hospital AKI (38% vs. 61%, *p* = 0.02) and severe or life-threatening bleeding (7% vs. 16%, *p* = 0.11).
Schäfer et al. [86]	166	3 German and 1 Danish centers	2.5/CP	Insertion pre-PCI (*n* = 68) vs. post-PCI (*n* = 98)	Pre-PCI insertion was associated with lower 30-day mortality rates (28% vs. 51%, *p* = 0.0039) and at multivariate regression analysis [HR 0.42, CI (0.21–0.82); *p* = 0.012].
**Extent of Revascularization in pts. With Mvcad**					
**Study**	**N° of pts.**	**N° of centers**	**Impella devices**	**Study groups**	**Main findings**
Aurigemma et al. [87]	152	17 Italian centers	2.5/CP	Pts with BCIS-JS RI < 0.67 vs. Pts with BCIS-JS RI ≥ 0.67	At 1-year FU, a more extensive revascularization (RI ≥ 0.67) was associated with better survival free of the composite of death, non-fatal MI, and non-fatal stroke (*p* = 0.006), mainly driven by significantly lower all-cause mortality (*p* = 0.005) pts. EF [HR: 0.96, CI (0.93–1.0); *p* = 0.05] and BCIS-JS RI < 0.67 [HR: 3.15, CI (1.2–5.8); *p* = 0.01) were the only predictors of the composite endpoint on multivariate analysis.
Lemor et al. [88]	198	57 US centers	2.5/CP	MV-PCI (*n* = 126) vs. CV-PCI (*n* = 72) (early MCS implantation)	In-hospital survival and rates of AKI were not significantly different between groups (69.8% MV-PCI vs. 65.3% CV-PCI; *p* = 0.51; and 29.9% vs. 34.2%; *p* = 0.64, respectively).
**Timing of Impella Insertion and Extent of Revascularization**					
**Study**	**N° of pts.**	**N° of centers**	**Impella devices**	**Study groups**	**Main findings**
Schäfer et al. [57]	202	3 German and 1 Italian centers	CP	CR (rSS ≤ 8; *n* = 130) vs. IR (rSS > 8; *n* = 72)	At 30-day FU, mortality was higher with post-PCI insertion (Impella post-PCI: 57%, Impella pre-PCI: 38%, *p* = 0.0053) and IR (rSS ≤ 8: 37%, rSS > 8: 56%, *p* = 0.0099). Patients with both pre-PCI Impella insertion and CR had a significantly lower mortality (33%) than those with IR and post-PCI insertion (72%, *p* < 0.001).

AMI; acute myocardial infarction; CS: cardiogenic shock; PCI: percutaneous coronary intervention; HR: hazard risk; CI: confidence interval; AKI: acute kidney injury; MV: multivessel; MCS: mechanical cardia support; CV: culprit vessel; BCSI-JS: British Cardiovascular Intervention Society myocardial jeopardy score (BCIS-JS); RI: revascularization index; CR: complete revascularization; IR: incomplete revascularization; FU: follow-up; rSS: residual Syntax Score.

## Data Availability

Not applicable.

## Abbreviations

CS	Cardiogenic shock
AMI	acute myocardial infarction
CAD	coronary artery disease
STEMI	ST-segment elevation MI
NSTEMI	non ST-segment elevation MI
PCI	percutaneous coronary intervention
MVD	multivessel disease
ACS	acute coronary syndromes
CLO	culprit lesion-only
MCS	mechanical circulatory support
IABP	intra-aortic balloon pump
ECMO	extracorporeal membrane oxygenation
AKI	acute kidney injury
